# Effect of Fly Ash on Mechanical Properties of Polymer Resin Grout

**DOI:** 10.3390/biomimetics8050392

**Published:** 2023-08-26

**Authors:** Ashraf A. M. Fadiel, Nuria S. Mohammed, Ahmad Baharuddin Abdul Rahman, Esam Abu Baker Ali, Taher Abu-Lebdeh, Florian Ion Tiberiu Petrescu

**Affiliations:** 1 Department of Civil Engineering, Omar Al-Mukhtar University, El-Bieda P.O. Box 919, Libya; ashraf.fadiel@omu.edu.ly (A.A.M.F.); nuria.saleh@omu.edu.ly (N.S.M.); 2 Department of Structure and Materials, Faculty of Civil Engineering, Universiti Teknologi Malaysia, Johor Bahru 81310, Johor, Malaysia; baharfka@utm.my; 3 Faculty of Mechanical Engineering, Universiti Teknologi Malaysia, Johor Bahru 81310, Johor, Malaysia; abaabesam2@graduate.utm.my; 4 Department of Civil, Architectural and Environmental Engineering, North Carolina A and T State University, Greensboro, NC 27411, USA; taher@ncat.edu; 5 “Theory of Mechanisms and Robots” Department, National University of Science and Technology POLYTECHNIC Bucharest, Splaiul Independentei Street 313, 060042 Bucharest, Romania

**Keywords:** biomaterials, polymer, resin grout, grout, mechanical properties, microstructure

## Abstract

High-strength grout is specified to increase the bond between grout and bar in grouted connections and to ensure that the forces in the bars can be transferred to the surrounding material accordingly. Although polymer grout is fast setting and rapid in strength development, the use of polymer mortar in grouted connections is still limited because of the lack of information and familiarity practitioners have regarding the product. The goal of this work is to investigate the mechanical characteristics and performance of polyester grout containing fly ash that can be used as an infill material for grouted connections. This study focused on the composition of polymer grout, which typically consists of a binder, hardener, and filler. In this particular case, the binder was made of unsaturated polyester resin and hardener, while the filler was fine sand. The aim of the research was to investigate the potential benefits of incorporating fly ash as an additional filler in polymer resin grout and examine the mechanical properties of polymer resin grout. To this end, varying amounts of fly ash were added to the mix, ranging from 0% to 32% of the total filler by volume, with a fixed polymer content of 40%. The performance of the resulting grout was evaluated through flowability, compression, and splitting tensile tests. The results of the experiments showed that, at a fly ash volume of 28%, the combination of fine sand and fly ash led to an improvement in grout strength; specifically, at this volume of fly ash, the compressive and tensile strengths increased by 24.7% and 124%, respectively, compared to the control mix. However, beyond a fly ash volume of 28%, the mechanical properties of the grout started to deteriorate. Due its superior properties in terms of compressive and flexural strengths over all examined mixes, the PRG-40-28 mix is ideal for use in the infill material for mechanical connections.

## 1. Introduction

The use of synthetic–organic polymers to replace part of the cement binder in concrete forms a concrete–polymer composite known as polymer-modified mortar (PMM). On the other hand, composites that utilize polymers and aggregates in the absence of cement are referred to as polymer mortar (PM) or polymer concrete (PC) [1]. PM employs fine aggregates, while PC employs both coarse and fine aggregates [2]. Polymer mortar or resin grout is a novel composite material with high mechanical strength and fast setting properties that can serve as a potential infill material for grouted connections [3,4,5,6]. The critical property required for an ideal grout is a strong anchorage bond, which is typically associated with high-strength grout [7]. Polymer mortars or resin grouts typically consist of a homogeneous mix of fine inorganic aggregates (sand), liquid resin, and curing agents, which cure at room temperature [8,9,10]. In addition, powder fillers play a significant role in enhancing the properties of polymer grout, such as gap-filling and mechanical strength. They can increase viscosity and decrease shrinkage by lowering the curing reaction rate and reducing the degree of exotherm [11,12]. Among the various fillers, fly ash is considered a crucial additive in polymer formulation due to its positive effects on mechanical strength. Researchers have conducted several studies on utilizing fly ash to improve the mechanical strength of polymer concrete. Fly ash can partially or fully replace ordinary river sand [13] and has been found to improve flexural strength when added to up to 75% by weight of fine aggregate. However, the flexural strength of polymer concrete declined at a higher level of fly ash and the concrete mix became unworkable. Soh et al. [14] compared the effects of different fillers, such as ground calcium carbonate, silica powder, and fly ash, on the mechanical properties of polyester mortars. Fly ash exhibited slightly higher strength than the other fillers, but the maximum limit of fly ash or ground calcium carbonate filler should not exceed 60% to obtain high-strength unsaturated polyester mortar. Similarly, Atzeni et al. [15] demonstrated that the specimens containing fly ash as filler in epoxy mortars exhibited higher compressive and bending strengths compared with quartz filler at shorter curing times. Enhanced mechanical properties for specimens containing fly ash are probably due to their greater fineness. The use of smaller filler sizes in polyester resin composites has been found to improve impact properties and strength due to the positive contribution of micro-fillers in the gaps between aggregate particles [16,17]. Although the use of powder fillers may enhance mechanical strength to a certain extent, excessive use of fillers beyond the desired value does not enhance the strength of polymer concrete [18].

The success of grouted splice connections is strongly dependent on grout characteristics. Typically, the most significant features required for an excellent grout are a high anchoring bond, which is typically linked with high strength grout; the ability to harden rapidly within predictable time; and minimization of construction delays. Typical cement grouts used in civil construction have several disadvantages, including a long curing period, gradual strength development, and reduced strength. Furthermore, a substantial amount of water is required in the manufacturing of cement grout to produce the desired flowability. This has an impact on the performance of the grout, such as a delayed initial setting time, a high rate of shrinkage, and a porous structure, which results in lower durability, sand particle sedimentation, the risk of bleeding, being less cohesive to the existing concrete surfaces, etc. [6]. Therefore, it is crucial to investigate the possibility of developing a high-performance grout. A relatively recent composite material that can be utilized as an infill material for grouted connections is polymer grout. This substance cures significantly more quickly than cement grout and has a strong compression strength. However, due to a lack of knowledge and familiarity with the product, the use of polymer grout in grouted connections is still rather restricted. To guarantee satisfactory performance, the use of grouting materials necessitates an understanding of fluid characteristics, the hardened properties of the grout materials, and the preparatory techniques to be applied on site. Consequently, this study was carried out with this objective in mind. Moreover, because sand’s specific weight is generally higher than that of the majority of substitute fillers, it has a propensity to settle during the hardening of polymer grout and cause nonuniformity in the finished product. As a result, it was decided to partially replace the sand with a micro-filler, namely fly ash. On the other hand, this study aims to reduce the negative impact of fly ash, which is frequently regarded as harmful to the environment, especially when it dumped in an open landfill.

Research on innovative grout, which is able to provide higher compressive strength and faster development of strength compared with conventional grout, is particularly essential for grouted connections between precast concrete structural elements. Connections such as beam splices, column splices, and beam-to-column connections depend on grouted connections for their structural integrity and stability. Cement grouts, which are usually used in construction for grouted connections, have a number of limitations, including long curing times, gradual development of strength, and lower compressive strength.

This study aims to investigate the mechanical and microstructure properties of polymer resin grout (PRG) using a fine aggregate, fly ash, unsaturated polyester resin as a binder, and MEKP as a hardener. This study adapts 40% of the polymer content and uses fly ash class F in a range from 0 to 32% of the total volume of the mix to enhance the performance of PRG. Earlier studies [13,19,20,21] investigated polymer concrete utilizing binder content in the range of 10 to 20%, but higher binder content is recommended when utilizing fine aggregates due to their high surface area [22,23]. The use of materials such as fly ash in polymer grout is an encouraging approach towards sustainability due to the utilization of waste and by-product materials [24,25,26,27,28]. Therefore, the objectives of this study are to investigate the effects of using fly ash on the mechanical properties of polyester resin grout and to obtain the optimum mix for producing a high-strength polymer grout.

## 2. Materials and Methods

### 2.1. Materials

In this section, we describe the constituent materials that make up PRG. These materials include resin, hardener, and filler materials such as sand and fly ash. We also discuss the properties of each material.

**Figure 1:**
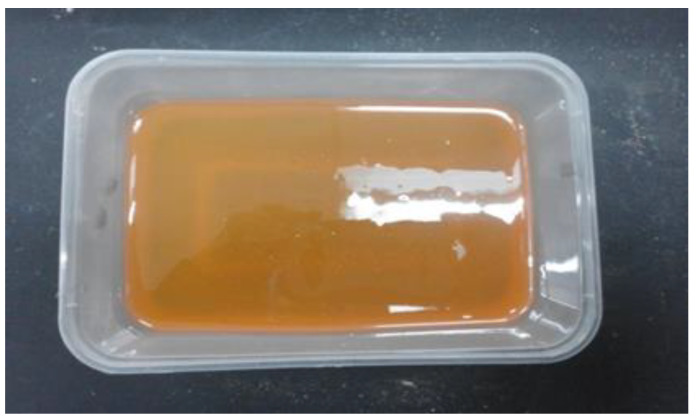
Unsaturated polyester resin used in this research.

Resin and Hardener: Polymer resin grout (PRG) was produced using an unsaturated polyester resin (Figure 1) as the main material and binder. The choice of this resin was based on its excellent mechanical properties, easy availability in the market, and comparatively lower cost than other types of thermoset resin. Table 1 provides a list of properties of the utilized unsaturated polyester resin. To catalyze the reaction, the polymer additive methyl ethyl ketone peroxide (MEKP) was added at a concentration of 1% of the weight of polyester resin. The unsaturated polyester resin and MEKP were purchased from Wee Tee Tong Chemicals Pte. Ltd., Singapore.

Filler: To produce the polymer resin grout (PRG), filling materials such as river sand and fly ash were utilized. The aggregate used was river sand with a density of 1600 kg/m^3^. Before being incorporated, the aggregate was subjected to oven drying at 100 ± 5 °C for one day to limit the moisture content of the filling material to 0–5%. Furthermore, the sand was sieved to 600 µm to obtain consistent particle size to ensure the consistency of prepared specimens.

Class F fly ash was utilized, which was obtained from a local power plant as a byproduct of coal-burning electric utilities. Fly ash’s particle sizes range from 0.3 to 250 µm, with the majority falling between 20 and 25 µm. With a density of 2500 kg/m^3^, the fly ash was used without any prior treatment. Fly ash was chosen due to its excellent compatibility with polyester resin and locally available materials. To ensure robust connections between the filler and binder (polyester resin), the fly ash was subjected to oven drying, which eliminated any excess moisture content and kept the moisture content below 0.5%.

### 2.2. Methodology

This study involved the preparation of nine different mixes of NPRG, namely PRG-40-0, PRG-40-4, PRG-40-8, PRG-40-12, PRG-40-16, PRG-40-20, PRG-40-24, PRG-40-28, and PRG-40-32. Fly ash was incorporated in the mixes at varying percentages ranging from 0% to 32% of the total mix volume, with increments of 4%, while maintaining a constant polymer content of 40%. Table 2 provides a summary of the mixture proportions. The aim of this experimental program was to assess the mechanical properties of non-cement polymer grout. The binder-to-filler proportion was held constant at 0.67, with unsaturated polymer resin being used as the binder at 40% of the total mix volume. To replace the primary filler material, river sand, fly ash was utilized within the range of 4–32%.

**Figure 2:**
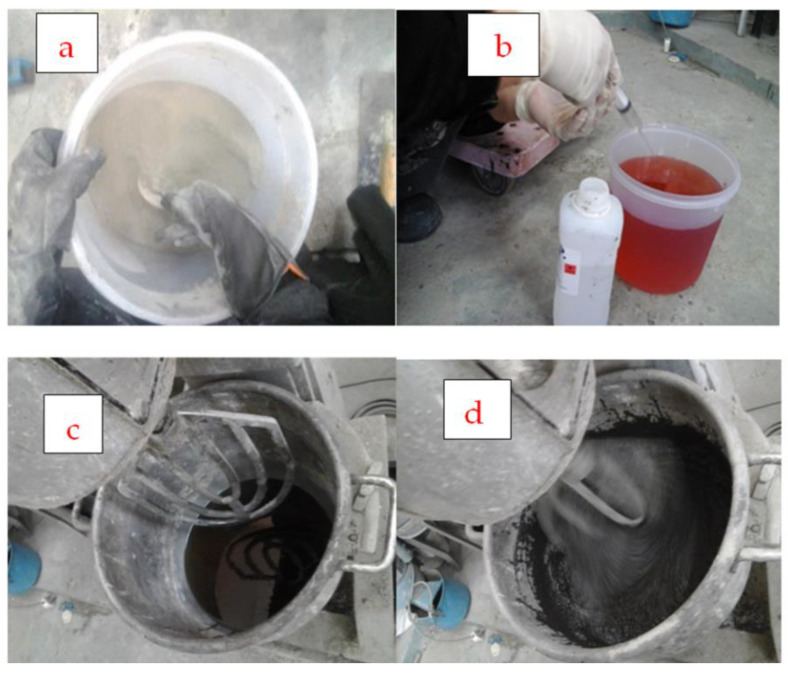
Preparation for polymer resin grout: (**a**) preparing the dry mix; (**b**) preparing the binder mix; (**c**) the binder mix poured into the mixer; (**d**) the dry mix poured into mixer and the mixed materials uniformly blended.

**Figure 3:**
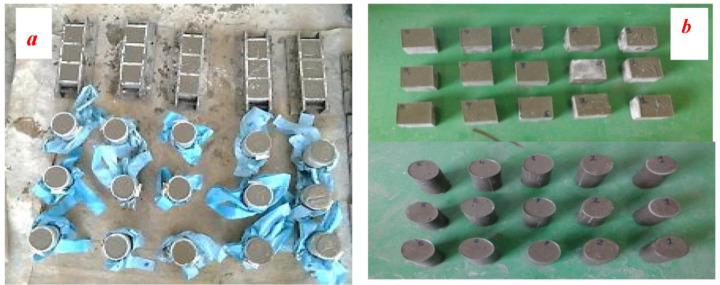
Preparation of specimens for mechanical properties tests: (**a**) PRG in molds; (**b**) PRG cubes and cylinders.

The dry mix of sands and fly ash was then added to the polyester resin along with the polymer additive at room temperature (21 ± 1), following the guidelines of JIS A 1181 (2005) [29], and mixed thoroughly in a mortar mixer until a homogeneous fresh polymer grout was obtained, as depicted in Figure 2. The sand and fly ash were prepared and weighed according to the mix proportion. They were subsequently mixed together for 1–2 min at a rate of 140 r/min until a uniform dry mix was obtained. Then, the liquid polyester resin was mixed with the hardener material for 3–5 min at a rate of 285 r/min. Once MEKP was added to the polyester resin, the color of the resin changed due to the polymerization process’s effect. Afterwards, the liquid mix was poured into the mixer, and the sand and fly ash were added to the liquid mix. The materials were mixed together until a uniformly blended mix of PRG was obtained. The resulting fresh polymer resin grout was employed to carry out the mortar flow spread test. Cube and cylinder specimens were cast without compaction, as illustrated in Figure 3.

### 2.3. Testing Methods

This study employed various testing methods, which included a flowability test that was carried out in accordance to with ASTM C230/C 203 M -04, a compression test using a universal compression test machine adhering to ASTM C 109—05, and a splitting tensile test using a MATEST universal testing machine (Treviolo, Italy) with a capacity of 2000 kN at a constant loading rate of 0.5 kN/s in accordance with ASTM C 469. Finally, the morphology images of PRG in this study were captured using scanning electron microscopy (SEM).

#### 2.3.1. Flowability Test

**Figure 4:**
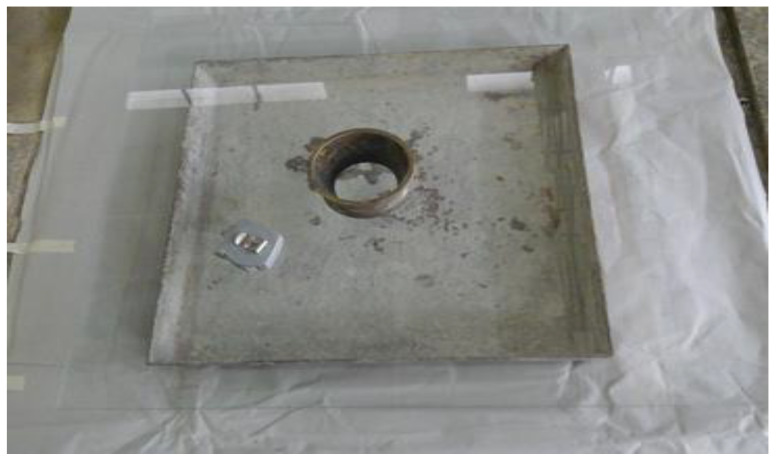
Test setup for flowability test.

To determine the flowability of the fresh polymer resin grout (PRG), the mortar flow spread test was employed. The standard flow mold, which adheres to the ASTM C 230/C 230M standard [30], was utilized to assess the flow spread of mortars, as depicted in Figure 4.

The mold cone utilized for the flowability test had an internal diameter of 50 mm and 100 mm, with a height of 70 mm. A glass plate was employed as a flow base. The mixture of polyester binder and filling material was poured into the cone, without any compaction during the test. The mold was subsequently raised vertically, enabling the PRG to flow over the glass plate. After the flow spread had completely ceased, the flow spread diameter was measured using a measuring tape. The flowability test setup is illustrated in Figure 4.

#### 2.3.2. Compressive Strength Test

Cube specimens with a cross-sectional size of 50 by 50 mm were employed for the compression test and were prepared using cube molds. The polymer resin grout compressive strength test was carried out using a universal compression test machine in compliance with ASTM C 109. The specimens were tested after one day without any curing. The compressive strength tests were performed utilizing a MATEST universal testing machine (Treviolo, Italy) with a capacity of 2000 kN. The test was performed at a constant loading rate of 1 kN/s and applied until failure of the specimen. The mean values of three specimens for each mix proportion were recorded.

#### 2.3.3. Splitting Tensile Test

Cylindrical specimens of size 50 × 100 mm (diameter × height) were prepared using a cylindrical mold. The specimens were demolded after 3 h of casting and tested after one day. The splitting tensile test was performed on the specimens using a MATEST universal testing machine (Treviolo, Italy) with a capacity of 2000 kN at a constant loading rate of 0.5 kN/s, in accordance with ASTM C 469. The average values of three specimens for each mix were recorded.

#### 2.3.4. Scanning Electron Microscope (SEM)

**Figure 5:**
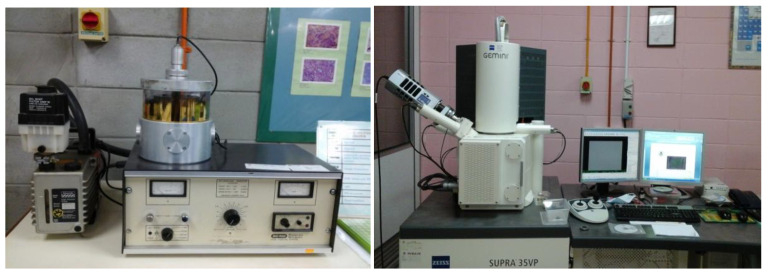
Test setup of the microstructure test.

The morphology of the RPG was examined using Zeiss Supra 35VP field electron scanning electron microscopy (FESEM). Figure 5 illustrates the experimental setup for the microstructure analysis. The microstructure examination involved placing a miniature specimen obtained from the crushed standard specimen on a coin using double cellophane, coating it with gold using a sputtering machine, and imaging the gold-sputtered RPG specimen using FESEM at a magnification of 1000 using the SE2 detector at 10 KV accelerating voltage. The working distance varied from 10.3 to 12.4 mm.

## 3. Results and Discussion

This study aimed to investigate the impact of partially substituting sand with fly ash on the flowability and strength of polyester resin grout with a total filler volume of 60%. This was performed to optimize the mix design of the material.

### 3.1. Flowability

**Figure 6:**
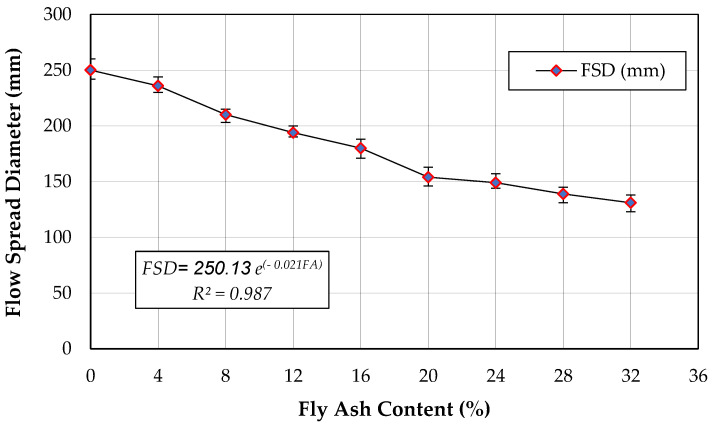
Flow spread of various fly ash contents.

To evaluate the workability of polymer grout, it is essential to measure its flowability, which plays a significant role in the infill material for grouted connections. This parameter governs the suitability of pouring the grout mix into the connection, making it a crucial aspect of grout mixes. Consequently, the grout mixes need to be workable throughout the grouting process. The flowability test results for different polymer resin grout mixes with varying fly ash contents are shown in Figure 6.

The relationship between flowability and fly ash content followed an exponential pattern (Equation (1)) with an impressive coefficient of determination of 0.99 (*R*^2^ = 0.99). Figure 6 depicts the flow spread diameter, which represents the flowability of the polymer resin grout. An increase in fly ash content resulted in a decrease in flow spread diameter, leading to a reduction in flowability of approximately 47.5% (from 250 to 131 mm) when 32% of the sand was substituted with fly ash. This decline in flowability was due to a decrease in bonding between the materials, with fly ash acting as a mobilized particle. As the fly ash volume increased, the mix volume also increased, leading to passive particle mobilization behavior [22]. Nonetheless, the flowability of the mix design was sufficient for the polymer grout, despite the decrease in flowability.

(1) $$FSD=250.13\;e^{-0.021\;FA}\;\;\;\;\;,\;\;R^{2}=0.99$$

where:

FSD: Flow spread diameter (mm),

FA: Fly ash content (%).

Flowability is a critical aspect of grout mixes, particularly for the infill material used in grouted connections. It determines the suitability of pouring the grout mix into the connection, making it a crucial factor to consider. Consequently, the grout mixes must be workable throughout the grouting process. These findings are helpful in selecting the appropriate fly ash content for use in PRG grout as infill material. All mixes achieved a flow spread diameter exceeding 130 mm. The mix that included 32% of fly ash had the lowest flow spread diameter at 131 mm. According to [31], a flow spread diameter of 130 mm is recommended for grouts used in splice connections.

### 3.2. Compressive and Splitting Tensile Strength

**Figure 7:**
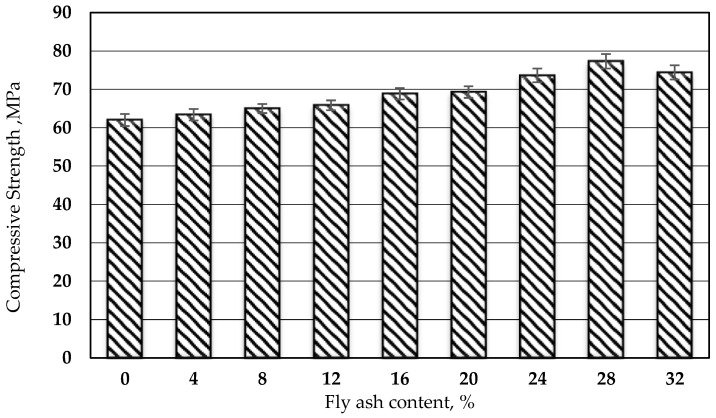
Compressive strength of polyester resin grout versus proportion of fly ash.

**Figure 8:**
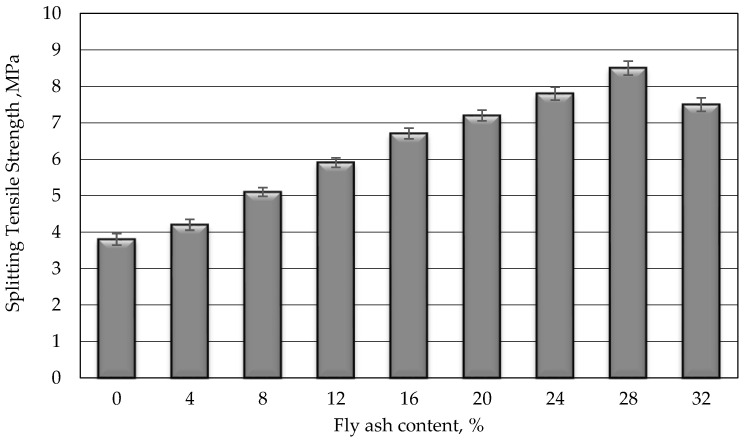
Tensile strength of polyester resin grout versus proportion of fly ash.

The compressive and splitting tensile strengths of polymer grout with varying fly ash contents are illustrated in Figure 7 and Figure 8. Both strengths showed a similar trend of increasing with the increase in fly ash content, up to 28%, beyond which the strength decreased. The compressive and tensile strengths increased by approximately 24% and 124%, respectively, compared to the control specimen (mix without fly ash). The excellent densely packed structure of the polymer grout, attributed to the special consistent shape of the fly ash particles, resulted in the elevation of both strengths at 28% of fly ash content. This particle shape effectively filled the gaps between the polymer grout materials. This was confirmed by a previous study by Duc. [32]. However, beyond 28% fly ash content, the interfacial bonding between materials weakened, causing the strength to decrease. This was evident from the reduced flowability at higher fly ash contents, as reported in [22].

The addition of the desired content of fly ash had a positive impact on the compressive strength of the PRG. However, exceeding the desired fly ash content led to a reduction in compressive strength. Similar findings have been documented in the literature [2,14], indicating that excessive fly ash beyond the optimum level can cause a decline in compressive strength.

The splitting tensile strength of PRG grout with 40% polymer binder content significantly increased with increasing fly ash content. The splitting tensile strength of PRG-40-28 grout attained the maximum strength of 8.5 MPa when the fly ash content was 28%. This splitting tensile strength obtained for PRG-40-28 was roughly 124% higher than the control grout, i.e., PRG-40-0 (0% fly ash). However, the splitting tensile strength decreased when fly ash content exceeded 28%.

**Figure 9:**
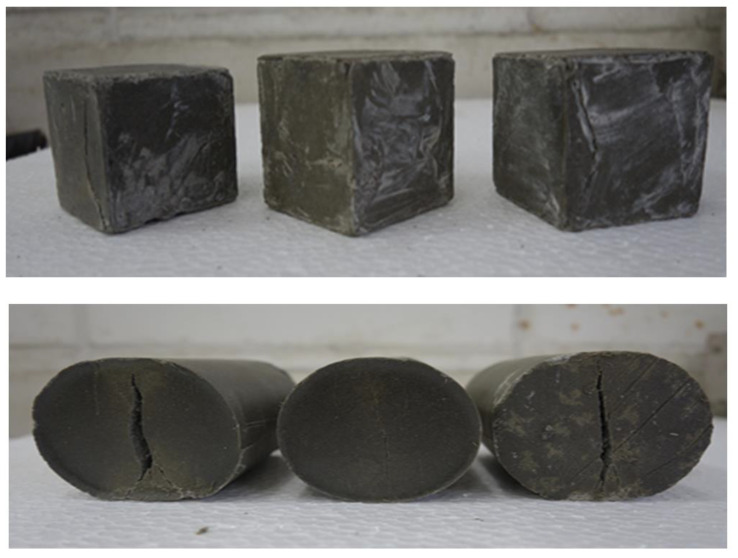
Modes of failure of PRG specimens due splitting and compression tests.

The observed mode of failure of the polymer resin grout exhibited a considerable change from fragile in PRG-40-0 to ductile failure in PRG-40-28, and then changed again to fragile in PRG-40-32, as shown in Figure 9.

The ratio of tensile strength to compressive strength (*f_t_*/*f_c_*) increased as the amount of fly ash increased up to 28%. The ratio (*f_t_*/*f_c_*) increased from 6.1% for the control mix (0% fly ash) to 11% for PRG that contains 28% fly ash. At 32% fly ash content, the tensile to compressive strength decreased to approximately 10%.

### 3.3. Microstructure Properties

**Figure 10:**
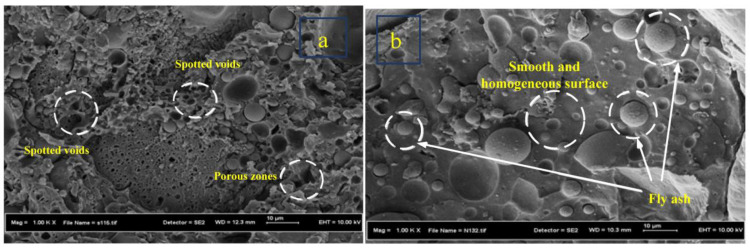
SEM micrographs of fracture surfaces of PRG specimens: (**a**) PRG-40-4; (**b**) PRG-40-24.

**Figure 11:**
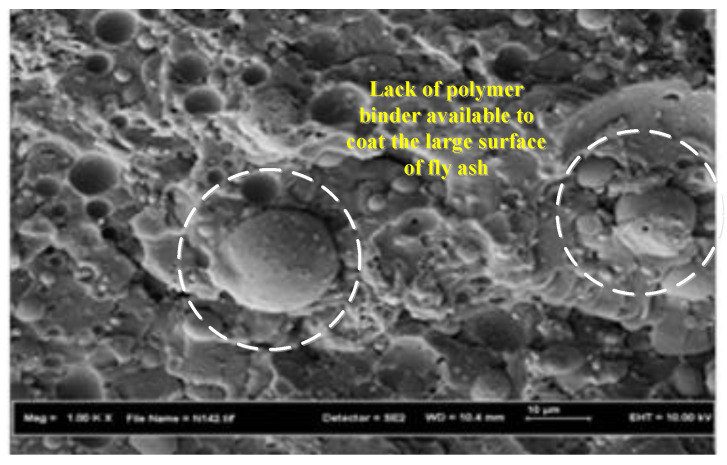
SEM micrographs of the PRG-40-32 mix.

This microstructure study was conducted to evaluate the effects of integrating fly ash on the microstructure properties of PRG and on its performance characteristics. The SEM microstructure of PRG-40-4, PRG-40-24, and PRG-40-28 is illustrated in Figure 10 and Figure 11.

As observed in Figure 10a, PRG-40-4 with 4% fly ash content was characterized by spotted voids and porous zones. The void presents the existence of a weak bond that can negatively affect the strength of the composite material.

Figure 10b depicts the FESEM image of the PRG-40-24 mix. From the visual observation of the fracture surfaces, it is noted that the specimen has a smooth and homogeneous surface, which is an indication of good bonding between the filler and the polyester matrix. This specimen is denser and less porous than the PRG-40-4 mix. The use of fly ash as a filling material contributes to reducing the internal voids between the sand particles, resulting in a dense packing structure [33,34]. In principle, the density of PRG contributes to its superior strength. Similar observations have been reported by other researchers. Gorninski et al. [35] reported that the different levels of strength are related to the number of pores in polymer concrete compounds. The high content of fly ash in polymer concrete improved the particle structure, leading to a decrease in porosity. The volume of voids in concrete influences its strength [28,29,30]. As the voids’ volume in the polymer mixture decreases, its strength increases [36,37,38,39].

Finally, Figure 11 shows the FESEM image of PRG-40-32 grout (32% fly ash) [40,41,42,43,44]. It can be noticed that the fly ash does not mix effectively with the polymer binder due to the lack of binder availability to coat the large surface area created using fly ash. Consequently, weak bonding between fly ash particles and the polymer binder developed in the composite, causing a decrease in the strength of the PRG grout. Similar behavior was observed in polymer concrete with high fly ash content, as reported by Varughese et al. [13].

## 4. Conclusions

This paper presented the findings of an experimental investigation into the compressive and splitting tensile strengths of PRG with varying fly ash content. The specimens were prepared using a consistent polyester resin dosage of 40%, while the fly ash content ranged from 0% to 32% of the total filler content, which was 60%. This study evaluated the impact of fly ash content on the flowability, compressive strength, and splitting tensile strength of PRG. The findings of this study are summarized as follows:

The addition of fly ash as a filler resulted in the PRG having superior compressive and splitting tensile strengths in comparison to the PRG without any filler.

Flowability assessments of the PRG indicate that PRG mixtures containing 40% polymer binder content and fly ash content up to 28% are appropriate for use as infill material for grouted connections.

Both compressive and splitting tensile strengths of PRG increased with the increase in fly ash content. However, if the fly ash content surpasses 28 percent, both compressive and tensile strengths decline.

Microstructural analysis revealed that the inclusion of fly ash in PRG-40-28 resulted in a denser matrix. This dense matrix facilitated the bonding capacity between NCPG materials, ultimately leading to the development of high strength.

This study indicates that the PRG-40-28 exhibits superior compressive and splitting tensile strengths, which can be attributed to its highly densely packed structure.

Polymer resin grout can be used as a bonding material for grouted sleeve connections. This paper presents the experimental results on the effectiveness of fly ash as a microfiller on the flowability, compression, and splitting tensile strengths of polymer grout that can be used as a potential infill material for grouted sleeve connections.

## Figures and Tables

**Table 1:** Properties of unsaturated polyester resin.

Property	Value
Styrene monomer content, (%)	39–44
Viscosity (Brookfield) at 25 °C, 60 rpm (cps)	538
Gel time, (min)	15
Barcol hardness	56
Density, (g/cm^3^)	1.1
Tensile strength, (MPa)	7

**Table 2:** Mixture proportions for different mixes.

Mix ID	Binder Content (Polyester Resin) (%)	Filler Content (Sand and Fly Ash) (%)	Fly Ash (%)	Sand (%)
PRG-40-0	40	60	0	60
PRG-40-4			4	56
PRG-40-8			8	52
PRG-40-12			12	48
PRG-40-16			16	44
PRG-40-20			20	40
PRG-40-24			24	36
PRG-40-28			28	32
PRG-40-32			32	28

## Data Availability

All the data selected and used in this paper are totally available.
